# Body Composition and Physical Fitness Profiles of Elite Female Japanese Wrestlers Aged <12 Years until >20 Years

**DOI:** 10.3390/sports8060081

**Published:** 2020-05-31

**Authors:** Hiroshi Arakawa, Daichi Yamashita, Takuma Arimitsu, Takashi Kawano, Takahiro Wada, Seshito Shimizu

**Affiliations:** 1 Department of Physical Education, International Budo University, Katsuura, Chiba 299-5295, Japan; 2 Department of Sport Science, Japan Institute of Sports Sciences, Kita, Tokyo 115-0056, Japan; daichi.yamashita@jpnsport.go.jp; 3 Department of Human Health, Hachinohe Gakuin University, Hachinohe, Aomori 031-8588, Japan; arimitsu@hachinohe-u.ac.jp; 4 Department of Physical Therapy, Touto Rehabilitation College, Meguro, Tokyo 153-0044, Japan; kawano4362@ybb.ne.jp; 5 Department of Physical Education, Kokushikan University, Tama, Tokyo 206-8515, Japan; wadatk@kokushikan.ac.jp; 6 Sports Design Lab, Minato, Tokyo 108-0023, Japan; shimizu@sportsdesignlab.com

**Keywords:** wrestling, body composition, muscle, strength

## Abstract

Studies evaluating the physical fitness levels of elite wrestlers during junior high school are limited. This study aimed to examine the body composition and physical fitness profiles of elite Japanese female wrestlers aged <12 years until >20 years. There were 114 elite female wrestlers enrolled. Measurements were conducted in the following age categories: <12 years (U-12), <15 years (U-15), <17 years (U-17: cadet), <20 years (U-20: junior), and >20 years (senior). Body composition variables consisted of body mass index (BMI), percent body fat, fat free mass, and fat free mass index (FFMI). Fitness measurements included grip strength, back strength, sit-up, rope-climbing, and endurance running tests. The wrestlers in this study demonstrated comparable or greater FFMI values (e.g., FFMI: 17.9 ± 0.4 kg/m^2^ for light and 19.8 ± 0.9 kg/m^2^ for heavy weight categories in U-20), when compared with young female wrestlers in previous studies, whereas stature, body mass, and BMI of the wrestlers in our study were unremarkable. Regarding the fitness assessment, a remarkable increase in back strength was observed after late puberty. An outstanding enhancement of muscle strength after late puberty, which is unlikely to occur in ordinary women, would be an important requirement to become the world’s top female wrestler.

## 1. Introduction

Wrestling has been a regular event in both ancient and modern Olympic Games. The ongoing international tournaments of wrestling encompass various age categories (i.e., schoolboys or schoolgirls: age <15 years, cadets: <17 years, juniors: <20 years, and seniors: >20 years) with sex and weight divisions. In general, enhanced physical fitness, including muscle strength, muscle power, muscle endurance, and cardiorespiratory endurance, is a fundamental requirement for wrestlers to be successful in high-level tournaments [1,2,3,4,5,6,7]. The physiological and anthropometrical tests could also be beneficial for young and senior wrestlers to optimize training programs and select talents effectively. 

To promote the effective use of the fitness tests, the anthropometrical and physiological profiles of successful athletes would be helpful as benchmarks. Several preceding studies have examined the body composition of elite wrestlers and proved that international-level wrestlers had greater fat free mass (FFM) and less fat tissue [2,8,9]. Regarding the physical capacities, a review and recent studies have indicated that the more successful wrestlers had greater muscular strength, power, and anaerobic capacity than the less successful wrestlers [3,8,10,11]. In contrast, although aerobic capability is assumed to be a basic requirement for wrestlers, there is a general agreement that it is not a defining factor of competitive success [2,3,10,11]. Aside from these studies, relatively little attention has been given to the physical profiles of elite wrestlers in the developmental stage. Although some studies have dealt with high school wrestlers [2,8,9,12,13], no study has ever compared, as is done for Judo [14], the profiles of very successful teenage wrestlers among different age categories.

Recently, the Japan Wrestling Federation (JWF) and the Japan Institute of Sports Sciences (JISS) conducted a nationwide measurement project on pubescent (elementary and junior high school children) and adolescent (high school children and minors) elite wrestlers (2012–2016). This project included the cross-sectional measurements of anthropometry, body composition, and physical fitness for each of the following age categories: <12 years (U-12), <15 years (U-15), < 17 years (U-17: cadet), and <20 years (U-20: junior). It should be also noted that Japanese female wrestling had one of the best performances at the Olympic Games in 2016 (four gold medals and one silver medal out of six weight categories) and the World Championships in 2017 (four gold medals, one silver medal, and one bronze medal out of eight weight categories). Furthermore, the younger categories of Japanese female wrestling had also successful achievements at the world championships of each age category. Therefore, identifying the physical fitness levels of elite female wrestlers in Japan will help the coaches and athletes understand the physiological characteristics of the strong athletes in the field. The purpose of this study was to examine the body composition and physical fitness profiles of elite Japanese female wrestlers from U-12 to senior age groups.

## 2. Materials and Methods

### 2.1. Study Design

This study was performed as a part of the Athlete Pathway Development Project (between 2012 and 2016), which was conducted by the JWF on commission of the Japan Sport Council. The JWF prepares the annual training camps that target adolescent elite Japanese wrestlers. The young elite wrestlers were selected nationwide based on the competition results; i.e., the inclusion and exclusion criteria for the study were to win either the gold or the silver medal at the national tournaments of each age category. The testing sessions were performed at these camps and conducted with the cooperation from the JISS.

All assessments were performed by experienced examiners who had practiced the prescribed training programs for data collection in JISS. All athletes and coaches were preliminarily informed in detail of the experimental procedures, possible risks, and benefits of the measurements. Each test was implemented upon receiving approval from all athletes and their parents with detailed informed consent. This study was approved by the institutional ethics committee (approval number: H26-034).

Each test consisted of laboratory and field measurements. The laboratory measurements included body composition (bioimpedance analysis) [15]. The field measurements consisted of isometric strength tests (back strength (BS) and grip strength (GS)), three sets of a 30 second incline sit-up test (IST), a rope-climbing test (RT), and six sets of a 300 m intermittent running test (300 m IRT). Each test is reported to have sufficient reliability and validity [15,16,17,18,19,20], except for the 300 m IRT [21].

The tests were completed within 1 day by all the athletes. At least two hours of rest was ensured between the 300 m IRT and the other fitness tests. All tests were performed during off-season, and no wrestlers were under a rapid weight-loss condition. Some participants performed the assessments more than once over several years. Accordingly, there is data duplication within the same wrestler in this study. In such cases, the latest results of the body composition and best performances of fitness measurements were used for further analyses. Regarding the IST and 300 m IRT, the best performances were determined by the total repetitions of three sets and the total time of six sets.

### 2.2. Participants and Categorization

Table 1 shows the age categories of the participants. As indicated, the numbers of wrestlers within each category (U-12, U-15, U-17, U-20, and the senior Japanese national team (NT)) were 25, 29, 19, 27, and 14, respectively. Wrestlers were assigned to light-weight (LW) or heavy-weight (HW) classifications based on their weight class. It should be noted that the specific weight class divisions vary from age group to age group. In this study, all classes in each age group were divided into halves based on an ordinal scale. Regarding the specific age category, which had odd-numbered weight classes, the median was assigned to LW.

In addition, the mean values of the four gold medalists at the Rio de Janeiro Olympics in 2016 were shown as a reference. The weight categories of the four champions were 48, 58, 63, and 69 kg. They were aged 23.0 ± 4.9 years on average when each measurement was obtained. The data of the two medalists of the lighter (48 and 58 kg) and the heavier (63 and 69 kg) classes were averaged and shown as the LW and the HW, respectively.

### 2.3. Body Composition

Body composition was assessed using the electric impedance technique (InBody 720, InBody Japan Inc., Tokyo, Japan) [15]. To minimize the effect of the circadian fluctuation of body fluid change on the measured value, all measurements were obtained in the early morning after micturition before breakfast. On the basis of the results measured by InBody 720, we calculated the body mass index (BMI) (kg·m^−2^) (body mass (BM) (kg) divided by stature squared (m^−2^), percent body fat (kg), and FFM (kg). Additionally, the FFM index (FFMI) (kg·m^−2^), which represents FFM (kg) divided by stature squared (m^−2^), was also calculated to indicate muscle mass standardized by stature.

### 2.4. Isometric Strengths

The maximal isometric BS [16] was measured using a back dynamometer (TKK5402, Takei Inc., Niigata, Japan). The length of the handle chain was adjusted to fit each participant, so that the angle of the participant’s trunk was at 30° of a right angle with their knees fully extended. The angle of 30° is considered a standard posture in this test [22]. This angle was endured with using a triangular ruler during the tests. The trials were conducted twice, and the better result was used in the analysis. The detailed testing procedures were based on the method described in the JISS measurement manual [22].

The maximal isometric GS [17] was also measured with a dynamometer (YX, Yagami Inc., Nagoya, Japan). The trials were performed twice for both left and right hand, and the better result for each side was used in the analysis. The detailed testing procedures were based on the method described in the JISS measurement manual [22].

### 2.5. Incline Sit-Up Test (IST)

The incline sit-up test (IST) [18] was used to assess strength, power, and local endurance of the musculature of front abdomen and hip-flexors. Before the measurement, the athletes were allowed to warm up and stretch on their own. Before the test, each wrestler lay on a sit-up bench inclined at 40°, and their feet were hooked under the ankle pads. During the test, the wrestlers performed sit-ups as quickly as possible with their knee angles kept flexed at 90°. No restriction was put on arm swings. The sit-up trials consisted of three sets of 30 s with 30 s intervals between the sets. During each repetition, the participants were instructed to adhere to the following two rules: to touch a bar that stands between the ankle pads, and to lower their trunk until their back touched the bench.

### 2.6. Rope-Climbing Test (RT)

The rope-climbing test (RT) [19,20] was used to assess the strength and power of the upper limb muscles, particularly regarding the pulling movement. Before the measurement, the participants were allowed to warm up and stretch on their own. For safety, this test was performed on a crash pad. Before the test, each wrestler sat on the crash pad with their legs straightened forward and both hands gripping the rope. They started climbing according to the examiner’s signal and climbed as fast as possible until they eventually touched the goal line. The goal line was located 4 m high from the upper surface of the crash pad. The rope was made of hemp. The time was measured by the examiner with a stopwatch. To ensure that the examiner could judge the finish time, the participants were instructed to exaggerate their actions of touching the goal line.

### 2.7. 300 m Intermittent Running Test

The 300 m IRT is a method of evaluating whole body endurance, which was designed by the Japan Wrestling Federation [21]. This test simulates the duration of a wrestling match and consists of six sets of 300 m sprints. According to the ongoing international rule, the matches were composed of a couple of 3 min periods with a 1 min interval. Given that the duration of a 300 m sprint is approximately 60 s, three sets of sprints with short breaks (10 s) roughly correspond to a single period of a wrestling match, as described in more detail by Chino et al. (2012) [21]. Although there is no study that examined reliability and validity of the 300 m IRT, Chino et al. indicated the usefulness of this test for an assessment of whole-body endurance for wrestlers.

This test was performed on a 400 m track. Examiners were allowed to have two stopwatches to measure the durations of the runs and the breaks, respectively. The wrestlers were instructed to try to achieve their best times of each set and to not preserve their energy for latter sets. In this test, three to four wrestlers were measured simultaneously as a group. Therefore, it should be noted that motivations might have mutually affected each other and influenced the running performance.

### 2.8. Statistical Analysis

Standard statistical methods were used to calculate the mean and standard deviations. Statistical significances were analyzed with R software (R version 3.3.3, R Core Team, Vienna, Austria) using the two-way analysis of variance (ANOVA) with the age group (five categories) and weight class (two classes) as factors. The Tukey’s test was used for the post-hoc analysis. The results of the post-hoc analysis for fitness tests are shown only between adjacent age groups to avoid confusion. The significance level was set at *p* < 0.05. In addition, the Cohen’s d effect size was also calculated for comparisons between the age groups. No statistical analysis of the four gold medalists at the Rio de Janeiro Olympics was performed because of the small sample size.

## 3. Results

The results of general physical sizes (stature, body mass, and BMI) are shown in Table 2. The statistical analysis revealed no significant interaction and significant main effects of age (*p* < 0.01) and weight class (*p* < 0.01) for all the variables. These effects indicate that each variable was larger in HW than in LW and increased with advancing age. The post-hoc analysis and the Cohen’s d effect size demonstrated that the three variables were significantly smaller in the U-12 group than in any other age group. There were no significant differences observed between the adjacent age groups older than U-15.

The results of the body composition assessments (body fat, FFM, and FFMI) are also shown in Table 2. The statistical analysis found no significant interaction and significant main effects of age (*p* < 0.01) and weight class (*p* < 0.01) for all the variables, indicating that all the variables were larger in HW than in LW and increased with advancing age. The mean body fat was the lowest in the U-12 group compared to the other age groups, regardless of the weight class (LW of U-12: 11.8% ± 3.4%, HW of U-12: 13.8% ± 3.8%). The post-hoc analysis demonstrated that there were no significant differences in body fat between the adjacent age groups older than U-15. Concerning FFM and FFMI, the Cohen’s d effect size demonstrated that the considerable increase with advancing age was observed within the comparisons between the older age groups (i.e., U-17 vs. U-20 and U-20 vs. Senior). As references, body fat, FFM, and FFMI of the four gold medalists (LW and HW) were 16.3 ± 1.1% and 20.4 ± 4.0%, 47.8 ± 5.9 kg and 51.5 ± 1.5 kg, and 18.6 ± 0.1 kg·m^−2^ and 20.1 ± 0.8 kg·m^−2^, respectively.

The results of the isometric strength tests are shown in Table 3. The statistical analysis for BS and BS/BM (BS per body mass) demonstrated significant interactions (BS: *p* < 0.01; BS/BM: *p* < 0.05) and significant main effects of age and the weight class. These main effects indicate that BS was stronger in HW than in LW, but BS/BM showed contradicting results, and it only increased with advancing age. Remarkably, the largest increases of BS and BS/BM were observed between U-20 and NT of HW (BS: from 113 ± 18 kgf to 161 ± 25 kgf, *p* < 0.01; BS/BM: from 1.75 ± 0.28 to 2.33 ± 0.29, *p* < 0.01). In addition, the Cohen’s d effect size demonstrated that there were considerable increases in BS and BS/BM with advancing age within the comparisons between older age groups (i.e., U-17 vs. U-20 and U-20 vs. Senior). Regarding the GS, the largest increase was observed between U-12 and U-15, regardless of the weight classes (GS in LW: from 18 ± 3 kgf to 27 ± 3 kgf, *p* < 0.01; GS in HW: from 23 ± 4 to 32 ± 4, *p* < 0.01). No significant age group effect was found for GS/BM. As references, BS and BS/BM of the four gold medalists (LW and HW) were 139 ± 4 kgf and 142 ± 31 kgf, and 2.42 ± 0.21 and 2.18 ± 0.31, respectively.

Table 4 shows the results of the other fitness tests. The two-way ANOVA demonstrated no significant interaction and significant main effects of age in the best (*p* < 0.01) and total repetitions (*p* < 0.01) of the IST. The Cohen’s d effect size demonstrated that the increases in total repetitions with advancing age were the largest between U-20 and NT for both weight categories (LW: from 66 ± 21 in U-20 to 84 ± 9 in NT, HW: from 63 ± 16 in U-20 to 75 ±11 in NT). Although no significant interaction and age group effect was observed for the results of the RT, the effect size demonstrated that there were considerable improvements with advancing age between U-20 and Senior in LW (d = 1.1), and U-17 and U-20 in HW (d = 0.9). Regarding the 300 m IRT, a significant main effect was found for the weight category, but not for the age group.

## 4. Discussion

There are extensive studies that examined the anthropometry, body composition, and physical fitness of successful adult wrestlers [4,10,23,24,25]. As for the younger age groups, although several studies have investigated the physical aspects of high school elite wrestlers [2,8,9,12], no study has evaluated elementary and junior high school elite wrestlers. This study is the first to examine the body composition and physical fitness parameters of very successful wrestlers in their early and middle teens.

Compared to preceding studies that investigated adolescent female wrestlers [9,12], the present elite wrestlers demonstrated no outstanding features concerning the stature, the body mass, and the BMI (Table 2, stature: 151.9 ± 3.8 cm in LW and 161.9 ± 4.6 cm in HW, body mass: 47.2 ± 2.5 kg in LW and 61.6 ± 5.8 kg in HW, BMI: 20.5 ± 1.1 in LW and 23.5 ± 2.3 in HW). When compared with athletes other than wrestlers [14,26,27,28,29], the present wrestlers did not have particularly large BMI (Table 2). Despite the importance of muscle development in wrestling, the present elite female wrestlers showed ordinary BMI, probably because competitive wrestling is conducted in a weight-class system. As for the comparison among age groups, there were significant increases in stature, body mass, and BMI only between U-12 and U-15 (Table 2). On the other hand, no significant increases were observed between adjacent age groups, except for between U-12 and U-15 (Table 2). These observations are in accordance with the general growth pattern of Japanese girls whose developments of body mass and BMI stop at approximately 15 years of age [30].

In this study, FFMI is the most useful measure for evaluating muscle tissue development (Table 2). According to Vardar et al. [12] who investigated female elite high-school-age wrestlers (age: 16.2 ± 1.1 years), the average stature and FFM were 1.62 m and 45.1 kg, respectively, yielding an FFMI of approximately 17.2 kg/m^2^. The results of the corresponding age group of the present study (Table 2) are approximately equivalent in LW (U-17: 17.0 ± 0.9 kg/m^2^) and exceeded in HW (U-17: 18.9 ± 1.2 kg/m^2^), when compared with those of Vardar et al.’s study population. On the other hand, other studies investigated slightly older female wrestlers and found that FFMI was 19.2 ± 2.5 kg/m^2^ in college wrestlers [31], and was 18.2 ± 0.8 kg/m^2^ in LW and 18.7 ± 1.4 kg/m^2^ in HW [9] in teenage elite wrestlers. In this regard, it should be noted that percent body fat can be estimated to be 1%–2% higher by bioimpedance analysis than by Dual energy X-ray absoaptiometry (DXA) [32]. Considering the discrepancy in the evaluated value between these methods, it can be interpreted that the present female wrestlers had comparable or slightly greater FFMI values than the female wrestlers in the previous studies. Besides, a few studies investigated the body composition of female young judokas who engaged in judo (judo is similar to wrestling in terms of grappling martial arts) [14]. Franchini et al. (2011) investigated national-level adolescent Spanish judokas. According to the results of percent body fat by the skinfold method [20], FFMI can be roughly calculated as 17.5 kg/m^2^ in cadets (16.5 ± 0.4 years) and 19.2 kg/m^2^ in juniors (18.6 ± 0.5 years). In addition, Santos et al. (2014) reported that FFMI of wrestlers and judokas (compounded) were 16.7 kg/m^2^ by DXA [26]. The female wrestlers in this study had a higher FFMI than the subjects of these previous studies [14,26].

In addition to body composition assessment, fitness tests were also conducted in this study. Focusing first on the isometric muscle strength (Table 3), the results of GS observed in the U-20 group are relatively close to those of Garcia-Pallares et al. (2012) who investigated teenage elite female wrestlers and found that the GS ranged from 27 to 35 kgf [9]. In contrast, the results of BS for NT and the gold medalists (Table 3) are larger than those reported by Garcia-Pallares et al. (2012), which ranged from 85 kgf to 116 kgf [9]. As for the comparison among age categories, the post-hoc analysis and the Cohen’s d effect size demonstrated that BS and BS/BM increased significantly with advancing age, even older than U-17 (Table 3), whereas there were no significant differences in GS and GS/BM between U-17 and NT. It should be noted in this regard that the stronger result of BS and BS/BM in NT compared to the other age groups (Table 3) might attribute the larger age interval between U-20 and NT (Table 1). Nonetheless, the findings of BS and BS/BM, including those of the gold medalists, suggest that successful Japanese female wrestlers built up muscles even after high school, especially around their back and hips. In general, the gain in muscle strength with advancing age in girls tends to stop earlier than that of boys [33]. The finding of the present study was, in contrast, that the improvement of muscle strength in the elite female wrestlers occurred during late and post puberty rather than early puberty. Therefore, it is inferred from the present results that the striking increase in muscle strength after late puberty, which is generally unlikely to occur in the normal female population, may be an important requirement to become the world’s top female wrestler. This inference suggests a continued training effect for muscle strength training for working athletes and coaches. Regarding the relationships between the muscle strength and the muscle size, the increase in absolute strength (BS) after puberty (Table 3) can be explained by the gain in muscle mass (i.e., FFM and FFMI, Table 2). In addition, the BS/BM also increased in this study after puberty (Table 3). This improvement in relative strength might attribute an effect of specific resistance exercise programs, such as the dead lift or the power clean. In this regard, however, we cannot discuss further because of the lack of details about the training status.

As in Table 4, we also examined the wrestling-specific fitness measurements (IST, RT, and 300 m IRT). Unfortunately, although previous studies have investigated the corresponding measurements in male wrestlers [21,23,25], no study has ever explored these measurements in female wrestlers. Thus, it is impossible to compare the present results with those of female wrestlers in previous studies. Regarding the comparison among age groups, although the post-hoc analyses found no significant differences among the adjacent age groups (Table 4), the effect size measures suggested the considerable improvements in performances particularly in late and post puberty. These results partly agree with the finding of Naka et al. [34] who reported that the upper-limb pulling power was significantly greater in the elite female Japanese wrestlers than in their sub-elite counterparts. Moreover, the results of effect size measures appear to be consistent with those of body composition (Table 2), in which the considerable gain in muscle mass with advancing age was indicated. Presumable rationales for the negative outcomes of the post-hoc tests are twofold; the small sample size and that these fitness tests use own body mass of each subject as a load. Specifically, the gain in body mass that occurs necessarily with growth is assumed to negatively affect the results of the IST, rope-climbing test, and 300 m IRT. It can be interpreted that the physical capacities have been steadily improved with advancing age, given that maintaining the performance of fitness tests while gaining in body mass contributes to an increase in positive mechanical work.

The present study has some limitations. Although the total sample size of the current study was 114, the sample size within each category was small (at least seven in LW of U-17 and in both LW and HW of senior). This limitation is not an issue of reliability but generalizability and bias. Second, this study used the electric impedance technique (InBody 720, InBody Japan Inc., Tokyo, Japan), which is not the gold standard for body composition assessment. Therefore, the present FFM, FFMI, and percent body fat results cannot be directly compared with those of previous studies using other methods, such as DXA. Third, the fitness tests were completed within 1 day by all the athletes. At least two hours of rest was endured between the 300 m IRT and the other fitness tests. However, some athletes might not have been able to perform their maximal performances because of fatigue. Finally, given that the present study was a cross-sectional investigation, the differences among age groups were only the comparisons between different subjects. A longitudinal investigation in a similar population will help in providing further support for our discussion on the comparisons among age groups.

## 5. Conclusions

The elite female adolescent Japanese wrestlers demonstrated comparable or greater FFMI values (Table 2), when compared with wrestlers in the previous studies [9,12,14,26], whereas the stature, the body mass, and the BMI were not remarkable. Regarding physical fitness assessments, the female wrestlers in this study demonstrated stronger back strength performance (Table 3) than the elite female wrestlers in the previous study [9]. A remarkable increase in back strength was observed after late puberty, when notable muscular strengthening is generally unlikely to occur in the normal female population. It can be inferred from the present results that an outstanding enhancement of muscle strength after late puberty may be an important requirement to become the world’s top female wrestler.

## Figures and Tables

**Table 1:** Summary of the participants in each category.

Items	Class	U-12	U-15	U-17	U-20	Senior (NT)
Number of subjects	LW	11	16	7	12	7
	HW	14	13	12	15	7
Mean age (years)	LW	11.0 ± 0.6	14.1 ± 0.9	16.3 ± 0.8	18.5 ± 1.2	21.4 ± 3.6
	HW	11.4 ± 0.5	14.4 ± 0.8	16.6 ± 0.5	18.6 ± 1.0	24.3 ± 5.6
Training hours (hours/day)	LW	4.0 ± 0.8	6.3 ± 1.3	6.5 ± 0.5	6.2 ± 0.4	-
	HW	5.5 ± 1.4	5.3 ± 2.1	5.5 ± 1.2	6.0 ± 0.0	-
Training frequency (days/week)	LW	2.2 ± 0.4	3.2 ± 0.7	3.5 ± 0.5	3.7 ± 0.5	-
	HW	2.3 ± 0.4	3.3 ± 1.5	3.7 ± 0.5	4.0 ± 0.5	-

LW: light-weight, HW: heavy-weight, NT: national team.

**Table 2:** Results of general physical sizes, including stature, body mass, body mass index (BMI), and body composition assessments, i.e., percent body fat, fat free mass (FFM), and fat free mass index (FFMI).

Items	Class	U-12	U-15	U-17	U-20	Senior(NT)	GoldMedalist
Stature (cm) ** ^##^	LW Cohen’s d	137.3 ± 5.0	149.8 ± 4.9 ^††^ 2.5	151.9 ± 3.8 0.5	154.2 ± 5.8 0.5	157.6 ± 4.9 0.6	160.0 ± 9.4
	HW Cohen’s d	150.3 ± 7.4	160.2 ± 5.5 ^††^ 1.5	161.9 ± 4.6 0.3	160.6 ± 2.7 0.3	163.6 ± 3.9 0.9	160.1 ± 1.1
Body mass (kg) ** ^##^	LW Cohen’s d	31.3 ± 2.4	43.9 ± 4.7 ^††^ 3.4	47.2 ± 2.5 0.9	51.5 ± 4.1 1.3	55.5 ± 4.4 0.9	57.1 ± 6.4
	HW Cohen’s d	41.5 ± 7.4	56.5 ± 5.0 ^††^ 2.4	61.6 ± 5.8 0.9	65.2 ± 6.1 0.6	69.0 ± 5.4 0.7	64.8 ± 5.2
BMI (kg/m^−2^) ** ^##^	LW Cohen’s d	16.6 ± 1.1	19.5 ± 1.6 ^††^ 2.1	20.5 ± 1.1 0.7	21.6 ± 1.0 1.0	22.3 ± 1.1 0.7	22.3 ± 0.1
	HW Cohen’s d	18.3 ± 1.8	22.0 ± 1.1 ^††^ 2.5	23.5 ± 2.3 0.8	25.2 ± 2.1 0.8	25.8 ± 1.4 0.3	25.3 ± 2.4
Body fat (%) ** ^##^	LW Cohen’s d	11.8 ± 3.4	15.1 ± 3.6 ^††^ 0.9	16.7 ± 4.0 0.4	17.4 ± 3.1 0.2	16.3 ± 2.3 0.4	16.3 ± 1.1
	HW Cohen’s d	13.8 ± 3.8	19.1 ± 4.5 ^††^ 1.3	19.4 ± 5.5 0.2	21.3 ± 4.7 0.4	20.0 ± 2.7 0.3	20.4 ± 4.0
FFM [(kg) ** ^##^	LW Cohen’s d	27.6 ± 2.4	37.1 ± 3.1 ^††^ 3.4	39.3 ± 3.4 0.7	42.5 ± 3.4 0.9	46.4 ± 3.4 1.1	47.8 ± 5.9
	HW Cohen’s d	35.6 ± 5.3	45.6 ± 4.4 ^††^ 2.1	49.5 ± 4.2 0.9	51.1 ± 3.0 0.4	55.2 ± 3.8 1.2	51.5 ± 1.5
FFMI (kg/m^−2^) ** ^##^	LW Cohen’s d	14.6 ± 0.8	16.5 ± 0.9 ^††^ 2.2	17.0 ± 0.9 0.6	17.9 ± 0.4 1.3	18.7 ± 0.7^(0.082)^ 1.4	18.6 ± 0.1
	HW Cohen’s d	15.7 ± 1.1	17.7 ± 0.8 ^††^ 2.1	18.9 ± 1.2^(0.079)^ 1.2	19.8 ± 0.9 0.8	20.6 ± 0.8 0.9	20.1 ± 0.8

LW: light-weight, HW: heavy-weight, NT: national team. The following symbols represent significant main effect or difference. **: *p* < 0.01, significant main effect of age category. ^##^: *p* < 0.01, significant main effect of weight class. ^††^: *p* < 0.01, significant difference with adjacent younger age categories. Cohen’s d values represent the effect sizes of difference with adjacent younger age categories.

**Table 3:** Results of the isometric strength tests (back and grip strengths).

Items	Class	U-12	U-15	U-17	U-20	Senior. (NT)	GoldMedalist
BS (kg) ^§§^ ** ^##^	LW Cohen’s d	65 ± 14	91 ± 12 ^††^ 1.9	86 ± 12 0.4	109 ± 17 1.6	123 ± 10 1.0	138 ± 4
	HW Cohen’s d	75 ± 9	90 ± 26 0.8	116 ± 17 ^††^ 1.2	113 ± 18 0.2	161 ± 25 ^††^ 2.2	142 ± 31
BS/BM ^§^ ** ^##^	LW Cohen’s d	2.08 ± 0.45	2.10 ± 0.32 0.0	1.85 ± 0.30 0.8	2.11 ± 0.23 1.0	2.23 ± 0.20 0.5	2.42 ± 0.21
	HW Cohen’s d	1.83 ± 0.29	1.60 ± 0.41 0.6	1.89 ± 0.22 0.9	1.75 ± 0.28 0.6	2.33 ± 0.29 ^††^ 2.0	2.18 ± 0.31
GS (kg) ** ^##^	LW Cohen’s d	18 ± 3	27 ± 3 ^††^ 3.3	28 ± 4 0.3	32 ± 6 0.9	-	-
	HW Cohen’s d	23 ± 4	32 ± 4 ^††^ 2.3	36 ± 5 0.7	36 ± 5 0.0	-	-
GS/BM ^#^	LW Cohen’s d	0.56 ± 0.05	0.62 ± 0.05 1.1	0.59 ± 0.06 0.4	0.62 ± 0.09 0.4	-	-
	HW Cohen’s d	0.56 ± 0.07	0.57 ± 0.07 0.2	0.58 ± 0.07 0.1	0.55 ± 0.07 0.5	-	-

BS: back strength, BS/BM: back strength per body mass, GS: grip strength, GS/BM: grip strength per body weight, LW: light-weight, HW: heavy-weight, NT: national team. The following symbols represent significant interaction, main effect, or difference. ^§^: *p* < 0.05, ^§§^: *p* < 0.01, significant interaction. **: *p* < 0.01, significant main effect of age category. ^#^: *p* < 0.05, ^##^: *p* < 0.01, significant main effect of weight class. ^††^: *p* < 0.01, significant difference with adjacent younger age categories. Cohen’s d values represent the effect sizes of difference with adjacent younger age categories.

**Table 4:** Results of the wrestling-specific fitness measurements.

Class	LW						HW					
Items	U-12	U-15	U-17	U-20	Senior(NT)	Gold Medalist	U-12	U-15	U-17	U-20	Senior(NT)	Gold Medalist
Incline sit-up test (IST) (times)												
1st set	25 ± 6	26 ± 4	29 ± 4	28 ± 6	32 ± 2	34 ± 1	24 ± 4	25 ± 5	28 ± 4	28 ± 4	31 ± 3	31 ± 1
2nd set	19 ± 5	18 ± 4	20 ± 4	21 ± 7	28 ± 3	30 ± 1	18 ± 5	16 ± 4	20 ± 6	20 ± 7	25 ± 4	23 ± 1
3rd set	15 ± 4	14 ± 4	15 ± 4	17 ± 9	23 ± 4	27 ± 1	15 ± 4	11 ± 5	14 ± 4	14 ± 6	20 ± 4	17 ± 1
Best repetitions ** Cohen’s d	26 ± 5	26 ± 4 0.1	29 ± 4 0.8	28 ± 6 0.1	32 ± 2 1.0	34 ± 1	24 ± 4	25 ± 5 0.2	28 ± 4 0.6	28 ± 4 0.1	31 ± 3 0.6	31 ± 1
Total repetitions ** Cohen’s d	60 ± 12	57 ± 11 0.2	63 ± 12 0.5	66 ± 21 0.2	84 ± 9 1.1	90 ± 3	57 ± 12	52 ± 13 0.5	62 ± 13 0.8	63 ± 16 0.0	75 ± 11 0.9	70 ± 1
Rope climbing test (RT) (s) (*p* = 0.058 for age) Cohen’s d		9.4 ± 1.3	10.0 ± 2.4 0.3	9.2 ± 2.3 0.3	7.7 ± 1.3 0.8	6.6 ± 1.3		9.9 ± 4.0	10.3 ± 2.3 0.1	8.4 ± 1.5 0.9	8.8 ± 1.6 0.3	7.7 ± 0.7
300 m IRT (s)												
1st set		57 ± 4	56 ± 5	56 ± 4	59 ± 3	57 ± 1		61 ± 4	58 ± 6	57 ± 2	61 ± 4	58 ± 0
2nd set		69 ± 5	70 ± 8	73 ± 7	70 ± 5	67 ± 3		76 ± 7	72 ± 7	74 ± 5	73 ± 6	73 ± 7
3rd set		75 ± 6	75 ± 7	78 ± 7	73 ± 5	72 ± 9		81 ± 8	76 ± 6	79 ± 6	77 ± 6	74 ± 6
4th set		72 ± 6	71 ± 7	73 ± 6	70 ± 6	68 ± 8		76 ± 8	73 ± 6	75 ± 4	74 ± 5	73 ± 6
5th set		79 ± 6	77 ± 6	81 ± 6	74 ± 3	71 ± 1		86 ± 11	80 ± 6	82 ± 5	78 ± 6	75 ± 2
6th set		77 ± 6	74 ± 5	77 ± 7	71 ± 4	69 ± 5		79 ± 7	77 ± 5	78 ± 5	75 ± 4	73 ± 4
Best set ^##^ (*p* = 0.076 for age) Cohen’s d		57 ± 4	56 ± 5 0.3	56 ± 4 0.1	59 ± 3 0.8	57 ± 1		61 ± 4	58 ± 6 0.6	57 ± 2 0.1	61 ± 4 0.9	58 ± 0
Total time ^##^ Cohen’s d		428 ± 28	423 ± 32 0.2	440 ± 32 0.5	417 ± 25 0.8	403 ± 25		459 ± 40	437 ± 33 0.6	445 ± 25 0.3	438 ± 26 0.2	426 ± 25

LW: light-weight, HW: heavy-weight, NT: national team. The following symbols represent significant main effect or difference. ^##^: *p* < 0.01, significant main effect of weight class. Cohen’s d values represent the effect sizes of difference with adjacent younger age categories.
